# Community-acquired methicillin-resistant *Staphylococcus aureus* invasive infections: a case series from Central-South Chile

**DOI:** 10.3389/fmed.2024.1365756

**Published:** 2024-05-15

**Authors:** Alejandro Aguayo-Reyes, Felipe Morales-León, Mario Quezada-Aguiluz, Martha Quezada, Sergio Mella, Gisela Riedel, Néstor Herrera-Chávez, Yohana Espine, Gerardo González-Rocha, Andrés Opazo-Capurro

**Affiliations:** ^1^Departamento de Medicina Interna, Facultad de Medicina, Universidad de Concepción, Grupo de Estudio de Enfermedades Infecciosas (GrEEn-UdeC), Concepción, Chile; ^2^Grupo de Estudio en Resistencia Antimicrobiana (GRAM), Universidad de Concepción, Concepción, Chile; ^3^Laboratorio de Investigación en Agentes Antibacterianos (LIAA), Facultad de Ciencias Biológicas, Universidad de Concepción, Concepción, Chile; ^4^Departamento de Farmacia, Facultad de Farmacia, Universidad de Concepción, Concepción, Chile; ^5^Sección Microbiología Celular y Molecular, Laboratorio Clínico Hospital Guillermo Grant Benavente, Concepción, Chile

**Keywords:** community-acquired infections, MRSA, ST8, molecular epidemiology, Chile

## Abstract

The emergence of community-acquired methicillin-resistant *Staphylococcus aureus* (CA-MRSA) infections at the end of the 20th century represents a significant shift in the epidemiology of staphylococcal infections and, consequently, their clinical management. There are diverse CA-MRSA clones that are widely spread worldwide, showing differences in their regional dissemination, which has been dynamically changing over time. Although the first CA-MRSA description occurred about 30 years ago, its epidemiology in certain regions, such as South America, has been poorly explored, resulting in a gap in the understanding of the epidemiology of CA-MRSA in under-represented countries/regions. This report describes the first four clinical cases of invasive infections caused by CA-MRSA in a tertiary hospital in the central-southern region of Chile. It also associates the clinical characteristics of the infections with the microbiological and molecular features of the isolates. The four *S. aureus* isolates belong to sequence type 8, which has been widely described as a cause of community-acquired infections. All of them presented a wide resistome and virulome. Additionally, in two of them, it was possible to reconstruct the COMER genetic element, present in the USA300-Latin American variant clone. Considering these findings, it is crucial to prepare for a potential increase in invasive CA-MRSA infections in Chile. This would involve enhancing current surveillance systems and maintaining a low threshold of suspicion for these infections among clinicians.

## Introduction

*Staphylococcus aureus* poses a significant challenge as a bacterial pathogen, causing a diverse array of infections in human medicine. In 2017, methicillin-resistant strains, designated as MRSA, have been designated as priority pathogens by the World Health Organization due to their considerable impact on public health (1, 2). MRSA strains were initially identified in 1961 within hospital settings associated to infections, and they were termed as hospital-acquired *S. aureus* (HA-MRSA) (3, 4). However, since the 1990s, novel MRSA clones designed as community-acquired (CA-MRSA) have emerged globally, which are distinct genetically from HA-MRSA, and importantly, they to affected healthy individuals in community settings (5).

Epidemiologically, the molecular analysis of MRSA strains unveils a multifaceted and evolving landscape, delineating distinct predominant genetic lineages present in different geographic regions and environmental contexts. Moreover, there exists a continual emergence of successful clones that replace previous lineages, observed across both hospital and community environments (6). Distinguishing themselves from HA-MRSA, infections stemming from CA-MRSA strains manifest in individuals previously deemed healthy and devoid of exposure to healthcare facilities. These infections frequently target specific demographics, encompassing pediatric cohorts, military personnel, men who have sex with men (MSM), participants in contact sports, inmates in prisons, among others (7).

In Latin America, CA-MRSA isolates were first described during the early 2000s (8). Currently, the population structure of CA-MRSA exhibits significant diversity, encompassing variants such as USA300, USA300-Latin American variant (USA300-LV), ST30-SCC*mec* IV, and ST5-SCC*mec* IV clones (3).

For decades in Chile, there persisted a pronounced dichotomy in the epidemiology of staphylococcal infections, characterized by a distinct prevalence of strains susceptible to antistaphylococcal penicillins in community-acquired infections. Conversely, HA-MRSA infections were associated to the predominant Chilean-Cordobes clone (ST5-SCC*mec* I) (9, 10). Despite the historical predominance of HA-MRSA clones in Chile, the first cases of infections caused by CA-MRSA in the country were documented in 2006 (11). Subsequently, the identification of CA-MRSA cases has exhibited a consistent rising trend, as indicated by data from the National Surveillance Program of the Instituto de Salud Pública de Chile (ISPCh).^1^

Despite the lack of comprehensive epidemiological investigations on CA-MRSA infections in Chile, infectious disease experts regularly encounter invasive infections originating from this pathogen, representing a novel challenge within clinical practice. Consequently, we present a case series of four infections caused by CA-MRSA strains in a tertiary hospital situated in the Central-South region of Chile. This series delineates the infection’s characteristics, clinical prognosis, the molecular epidemiology, and molecular features of the isolates.

All cases that fulfilled the epidemiological criteria for CA-MRSA infection were identified from the local hospital registry of staphylococcal bacteremias. Following this, comprehensive clinical records were extracted from the electronic database of the hospital. Then, species identification and susceptibility tests were carried out by the automatized platforms Vitek MS (bioMérieux®) and Vitek 2 (bioMérieux®), respectively. Afterwards, the four CA-MRSA isolates underwent whole-genome sequencing (WGS) at SeqCenter Inc.^2^ utilizing the Illumina NextSeq 2000 platform. After sequencing, the raw reads generated were assembled using SPAdes v3.15.4. Following this, the SCC*mec* and Spa-type of the isolates were determined using the SCCmecFinder v1.2 tool^3^ and spaTyper v1.0^4^ provided on the Center for Genomic Epidemiology (CGE) server.

Additionally, the sequence types (STs) of the isolates were determined using the MLST tool accessible on the Galaxy Australia platform.^5^ Regarding the screening for resistance and virulence genes, ABRicate v1.0.1 was employed, utilizing the NCBI Bacterial Antimicrobial Resistance Reference Gene Database for resistance genes and the Virulence Factor Database (VFDB) for virulence genes.

Besides, genes related to copper and mercury resistance (COMER) and arginine catabolic mobile (ACME) elements, specifically *copA, argR*, *arcABCD*-locus, *speG* genes for COMER, and *merABR* locus, *mco, lysR, abi*, MFS genes for ACME elements, were identified via BLAST analysis. Subsequently, these elements were reconstructed *in silico* utilizing the Geneious Prime software (version 2023.2.1) from BiomattersⓇ. The genomes of *S. aureus* CA12 (GenBank accession number CP007672) and USA300 FPR3757 (GenBank accession number CP000255) were used as references for this process.

Furthermore, a phylogenetic tree was constructed, incorporating ten genomes of MRSA ST8 isolates from Chile, previously deposited in the Pathogen Watch database.^6^ Additionally, genomes of the ST8-USA300 (accession number NKCW01000010.1) and ST8-USA300-LV (Latin American variant, accession number CP007672.1) clones were included as reference sequences. Finally, the phylogenetic tree was constructed by aligning the core genome of the isolates using Roary v3.13.0, followed by the extraction of single nucleotide polymorphisms (SNPs) utilizing the SNP-sites tool (12). Subsequently, IQ-Tree was employed to generate the tree, and the final visualization and editing were performed using the interactive Tree of Life (iTOL) tool (13).

## Case 1

**Figure 1:**
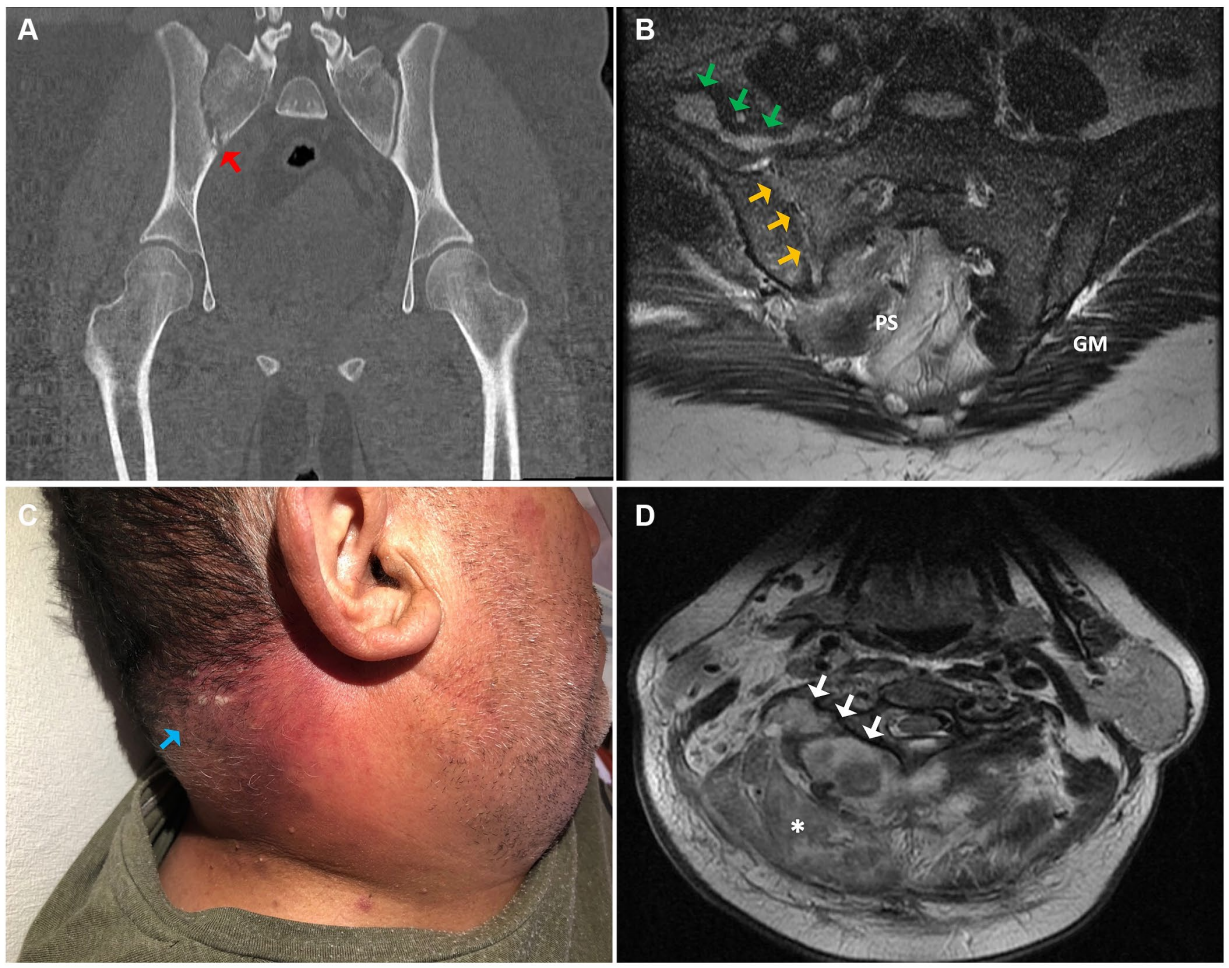
**(A)** Coronal section of the pelvic computed tomography (CT) scan, showing bone erosions and fragmentation of the right sacroiliac joint (red arrow). **(B)** Axial section of the pelvic (T2-weighted MRI) evidencing synovitis, osteitis, and erosions of the right sacroiliac joint (yellow arrows) in continuity with periarticular collections (green arrows). **(C)** An erythematous plaque with multiple pustules in the posterior cervical region concordant with staphylococcal anthrax (light blue arrow). **(D)** A deep right paravertebral cervical abscess is observed on the axial T2-weighted MRI (white arrows), associated with increased soft tissue signal congruent with a phlegmon (asterisk). PS, presacral space; GM, gluteus maximus.

A 26-year-old patient, overweight, presented at the emergency department with a three-day history of progressively worsening lower back pain accompanied by febrile symptoms. Additionally, the patient reported difficulty walking, with no other notable symptoms. Upon admission, the patient exhibited a fever (38.2°C) and tenderness in the lumbosacral and right gluteal regions upon palpation. Limited hip flexion on the ipsilateral side was also noted. Subsequently, a computed tomography (CT) scan was performed, revealing heterogeneous enhancement of the right psoas-iliac region, along with inflammatory changes in the surrounding tissues and erosion of the right sacroiliac joint (Figure 1A).

Upon admission, a blood chemistry panel, complemented with a complete blood cell count and blood cultures, was conducted, revealing the presence of Gram-positive cocci bacteria after 13.5 h of incubation (Table 1). Then, empirical antibiotic therapy with cefazolin was promptly initiated and later replaced by vancomycin upon confirmation of a MRSA isolate. For a more precise assessment of the lesions, a magnetic resonance imaging (MRI) study was performed, confirming acute right sacroiliitis and collections in the psoas-iliacus muscle, right gluteus maximus, and right paravertebral musculature (Figure 1B). Additionally, echocardiographic assessment revealed no signs of infective endocarditis. Subsequently, the patient underwent a six-week course of vancomycin therapy, followed by another six weeks of oral treatment with linezolid, resulting in complete resolution of the lesions.

**Table 1:** Clinical characteristics and laboratory parameters of the patients with infections caused by CA-MRSA.

Case	Primary site (infection)	Blood cultures at admission	Follow up blood cultures	WBC (x10^3^/μL; 4–10)	CPR (mg/dL; <1.0)	PCT (ng/mL; <0.05)	Lactate (mg/dL; 4.5–19.8)	SCr (mg/dL; 0.7–1.3)	Antibiotic therapy	Outcome
1	Osteoarticular	Positive	Negative	18.4	33.2	0.45	5.8	1.22	Vancomycin 6 weeks followed by linezolid 6 weeks	Alive
2	Skin/soft tissue	Positive	Positive	28.2	28.9	-	14.5	1.19	Vancomycin 6 weeks followed by linezolid 6 weeks	Alive
3	Lung	Positive	-	8	13.1	44.95	48.4	1.09	Vancomycin	Dead
4	Lung	Positive	Negative	2.6	17.5	48.49	30.8	1.72	Vancomycin	Dead

WBC, white blood cell count; CPR, C protein reactive; PCT, procalcitonin; SCr, serum creatinine. Normal values are indicated in parenthesis.

Additionally, the molecular analysis of the CA-MRSA isolate (008UDEC) recovered from this patient revealed that it belongs to the ST8 and the presence of the SCC*mec* IVb element. Notably, this isolate lacked the ACME and COMER elements, as indicated in the Supplementary Figure S1. In addition to the *mecA* gene found within the SCC*mec* element, the 008UDEC isolate contained the *fosB* and *tet* (38) genes, conferring resistance to fosfomycin and tetracycline, respectively. Of particular interest, within its extensive virulome, this isolate was distinguished by the presence of the *eta* gene, encoding an exfoliative toxin (14), which may enhance the virulence potential of this strain.

## Case 2

A 55-year-old male patient, with a history of coronary artery disease, type 2 diabetes mellitus, and obesity, presented at the emergency department with a 7-day history of pain, swelling, and redness in the posterior cervical region, accompanied by feelings of feverishness and severe malaise. These symptoms gradually developed after a superficial lesion with scissors at a barbershop. Additionally, he experienced sudden-onset dyspnea and retrosternal pleuritic pain, prompting him to seek medical attention. Upon physical examination, an erythematous cervical lesion was observed, highly tender upon palpation, exhibiting local warmth and multiple pustules (Figure 1C).

Subsequently, the patient was admitted to the surgical unit with a diagnosis of cervical abscess. Blood cultures were obtained whereas empirical antibiotic therapy with ceftriaxone and metronidazole was initiated. Initial investigations indicated the absence of ischemic changes on electrocardiography, along with a notable elevation in blood inflammatory markers (Table 1).

Due to pleuritic chest pain exacerbated by inspiration, a neck and chest CT angiography was conducted, unveiling thrombosis of the right internal jugular vein. Additionally, inflammatory changes were observed in the right cervical and parotid muscle planes, and a confirmed diagnosis of right basal segment pulmonary embolism was made, suggestive of Lemierre-like syndrome. Subsequently, blood cultures yielded positive results after 11 h, indicating the presence of Gram-positive cocci in clusters. Consequently, the antibiotic regimen was modified to cefazolin and later changed to vancomycin following confirmation of MRSA growth in the final culture.

Furthermore, a cervical spine MRI confirmed the presence of a paravertebral posteromedial cervical abscess with posterior epidural intraspinal extension, paravertebral muscle edema, and a small prevertebral collection (Figure 1D). Subsequently, surgical drainage was performed, yielding abundant pus emanating from the paraspinal muscles and epidural space, with evident macroscopic involvement of the bone. Cultures obtained from the drainage revealed the growth of a MRSA isolate. Biopsy results indicated active chronic inflammation and focal necrosis of the muscle tissue. The patient underwent a six-week course of intravenous therapy with vancomycin, followed by an additional six weeks of oral linezolid, resulting in a successful clinical outcome.

Furthermore, molecular analysis of the CA-MRSA strain collected (014UDEC) indicated that it belongs to the ST8, akin to the previous case. Additionally, this isolate harbored the SCC*mec* IVb cassette and the COMER element, as detailed in the Supplementary Figure S1. Similarly to the 008UDEC isolate, 014UDEC possessed the fosfomycin-resistance gene *fosB* and the tetracycline-resistance gene *tet* (38), alongside the *mecA* gene found within the SCC*mec* cassette. Furthermore, it exhibited a diverse virulome, encompassing numerous genes implicated in adhesion, immune system evasion, siderophores, and exotoxins/exoenzymes, as outlined in the Supplementary Figure S1. Interestingly, this isolate was not typeable by the *spa* typing scheme.

## Case 3

**Figure 2:**
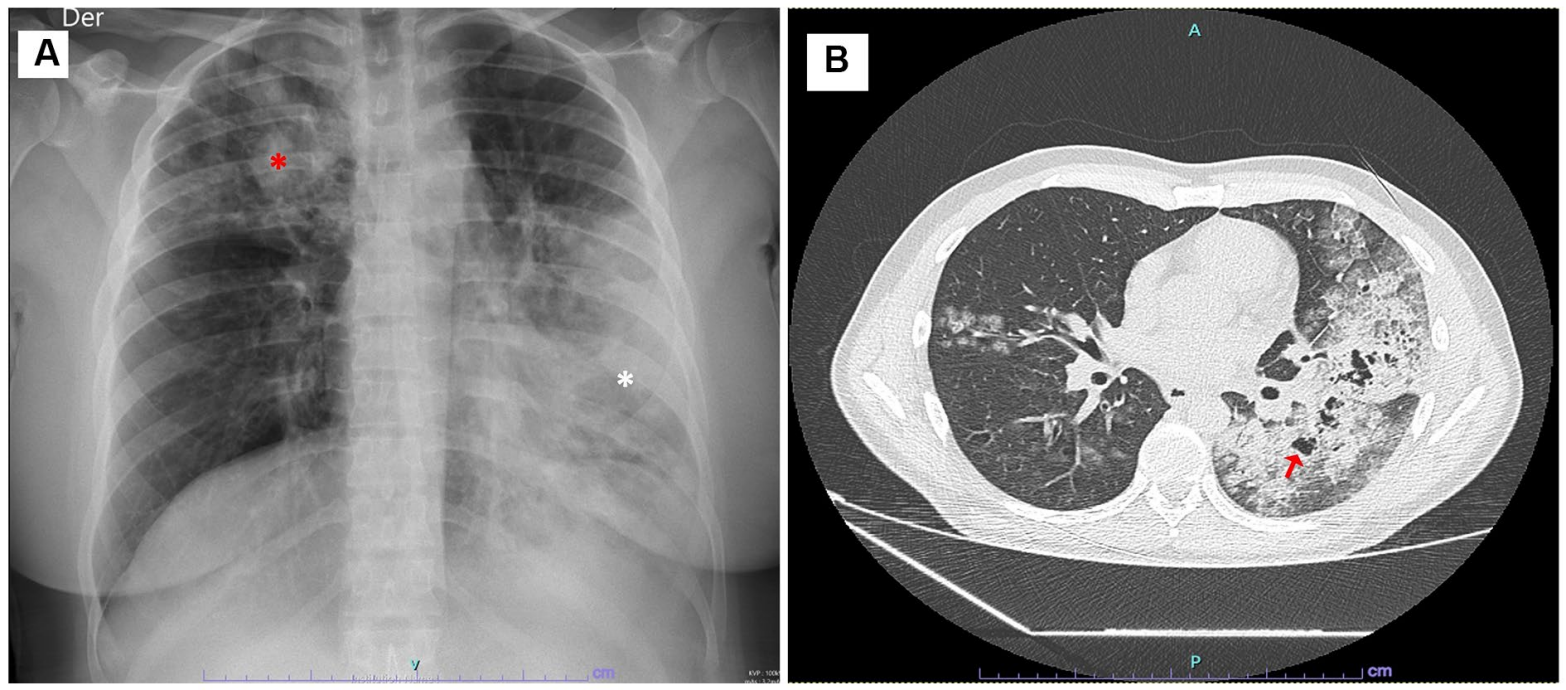
**(A)** Chest X-ray image revealing a consolidative opacity throughout the entire left lower lobe (white asterisk) and multiple alveolar filling opacities in the upper right lobe (red asterisk). **(B)** Chest computed tomography (CT) scan revealing consolidated and ground-glass opacities in the left lower lobe with the presence of pneumatoceles (red arrow), consistent with necrotizing pneumonia.

A 27-year-old female patient with a history of untreated asthma, who was incarcerated, was brought to a tertiary hospital from the prison due to a five-day history of fever, myalgias, asthenia, and adynamia, accompanied by worsening respiratory distress over the last 24 h. Upon arrival at the emergency department, she was conscious but severely dyspneic, with an oxygen saturation of 86% while receiving additional oxygen supplementation via low-flow nasal cannula (FiO_2_: 0.4). Furthermore, her heart rate was 123 beats per minute, and her body temperature was 37.6°C. Physical examination revealed crackles in the left lung base and diffuse wheezing. Following admission, she received high-flow oxygen therapy (FiO_2_: 1), parenteral corticosteroids, and bronchodilator therapy. Moreover, a portable chest X-ray confirmed multifocal pneumonia (Figure 2A).

Additionally, respiratory-virus molecular testing, along with blood and sputum cultures, were conducted, while empirical antibiotic therapy with piperacillin-tazobactam was initiated. However, the patient’s condition rapidly deteriorated, necessitating mechanical ventilation and transfer to the intensive care unit (ICU). Due to her clinical instability, a chest CT could not be performed. Baseline laboratory tests are summarized in Table 1, confirming influenza B infection, prompting the addition of oseltamivir to her treatment regimen. Given her severe respiratory distress, extracorporeal membrane oxygenation (ECMO) support was initiated. After 14 h of incubation, blood cultures yielded positive results, indicating the presence of Gram-positive cocci in clusters. Consequently, the empirical antibiotic regimen was adjusted to include vancomycin. Both blood cultures and sputum cultures were positive for MRSA (vancomycin minimum inhibitory concentration [MIC] ≤ 0.5 μg/mL). On her second day in the ICU, the patient also required high-volume hemofiltration. Unfortunately, in the subsequent days, the patient’s condition deteriorated further, culminating in multiple organ failure, and the patient died on the fifth day of hospitalization due to systemic failure.

Molecular analysis of the CA-MRSA strain (005UDEC) recovered from the patient revealed that it was part of the ST8 lineage, as detailed in the Supplementary Figure S1. Additionally, it was found to harbor the SCC*mec* IVb cassette containing the *mecA* gene. Moreover, this strain contains the COMER element, while ACME was found to be absent. In terms of antibiotic resistance genes, the resistome of this strain is larger compared to other isolates included in this case series, as it also contains the aminoglycoside resistance genes *aph (2″)* and *aph (3′)-IIIa*.

## Case 4

A 41-year-old male patient, with a history of active smoking and no other chronic conditions, presented with a one-month history of a cutaneous abscess in the left gluteal region. Despite undergoing different antibiotic regimens and wound care, the abscess did not show improvement.

The patient requested medical attention at a tertiary hospital due to a five-day history of cough, progressive dyspnea, myalgias, profound malaise, and, on the last day, hemoptysis. Upon admission to the hospital, the patient presented with severe respiratory failure, hypotension, and signs of poor perfusion, necessitating noradrenaline support and supplemental oxygen via high-flow nasal cannula. Furthermore, blood cultures were obtained immediately upon admission to the intensive care unit (ICU), where the patient was promptly intubated. Initial empirical antibiotic therapy comprised a single dose of piperacillin-tazobactam, followed by administration of vancomycin and imipenem-cilastatin. Baseline laboratory tests are detailed in Table 1.

Additionally, a chest CT scan unveiled extensive necrotizing pneumonia (Figure 2B). Subsequently, a bronchoalveolar lavage was conducted, uncovering active bleeding from the left lower lobe and highly inflamed bronchial mucosa. The patient’s condition continued to deteriorate, characterized by coagulopathy and oliguria, necessitating the addition of a second vasoactive drug to achieve the macro hemodynamic goals. Additionally, hemofiltration was initiated.

Relevantly, an influenza B co-infection was confirmed, and a preliminary report from the microbiology laboratory indicated the presence of Gram-positive cocci in peripheral blood cultures. Consequently, oseltamivir was added to the treatment regimen, while antibiotic therapy remained unchanged. Both bronchoalveolar lavage and blood cultures confirmed the growth of MRSA. Following evaluation by the infectious diseases team, vancomycin and oseltamivir were continued, while the imipenem-cilastatin treatment was ceased. Additionally, plasma vancomycin levels were monitored, and adequate pharmacokinetics-pharmacodynamics targets were achieved. Transthoracic echocardiography showed no signs of infective endocarditis, and fundoscopy revealed normal findings. Unfortunately, despite the therapeutic measures implemented, the patient developed multiple organ failure and died on the fourth day of hospitalization.

In this case, molecular analysis of the CA-MRSA strain (004UDEC) showed that it belongs to the ST8 and carries the *mecA* gene within an SCC*mec* IVb cassette. Notably, the strain lacked both the COMER and ACME elements (Supplementary Figure S1). Furthermore, it harbored the *fosB* and *tet* (38) genes, similarly to the other isolates included in this study. Additionally, virulence genes detected in 004UDEC included those involved in adhesion, immune system evasion, siderophores, and exotoxins/exoenzymes (Supplementary Figure S1).

## Discussion

For many years in Chile, the traditional dichotomy in staphylococcal infections persisted, wherein the presence of MRSA was primarily observed in healthcare facilities, characterized by the dominance of the Chilean-Cordobes clone (ST5/SCC*mec* I) (11, 15). In contrast, community-acquired infections were usually caused by methicillin-susceptible *S. aureus* (MSSA) strains. However, this duality is apparently changing associated to the emergence of CA-MRSA infections recently described in the country (11, 15).

In this regard, the implementation of a surveillance program by the ISPCh has revealed a rising trend in local cases of MRSA infections. Despite the absence of robust data, current evidence reveals a diversification of MRSA lineages in Chile, encompassing both hospital-acquired and community-acquired infections, mirroring trends observed in other countries (9).

Considering the aforesaid context, the clinical cases described here demonstrate all the classic clinical characteristics associated with CA-MRSA infections, such as patients with no history of contact with healthcare facilities, no prior antibiotic usage, absence of invasive medical procedures, typically involving young individuals with low associated morbidity. Furthermore, one of the cases affects a frequently identified risk group: individuals residing in prisons (16). Furthermore, the four cases were characterized by severe clinical symptoms, notably complicated skin and soft tissue infections, osteomyelitis, and necrotizing pneumonias. These clinical presentations align strongly with those widely described in the medical literature for CA-MRSA infections (7, 17).

Notably, both fatal cases described in our study were associated with patients who had a concomitant viral influenza co-infection. This phenomenon increases the likelihood of a secondary pulmonary infection by colonizing *S. aureus*, which occurs due to the damage inflicted on the respiratory epithelia by the influenza virus, thereby facilitating the subsequent spread of colonizing *S. aureus* from the nasal cavity to the lungs (18, 19).

The increased virulence observed in these strains is likely the consequence of a multifaceted interplay of multiple factors, therefore, it cannot be solely attributed to the presence or absence of any specific genetic determinant. Instead, it is likely influenced by a combination of genetic, environmental, and host-related factors, contributing to the overall pathogenicity of the strains (7). In this context, following the rise of CA-MRSA, it has been proposed that the presence of the Panton-Valentine leukocidin (PVL) might contribute to the increased virulence of these isolates. However, while PVL has consistently been epidemiologically associated with community clones, its sole presence does not fully account for the heightened severity of clinical presentations caused by CA-MRSA (3, 20).

Alarmingly, the potential impact of CA-MRSA infections is notable, especially in non-industrialized nations like Chile. In this sense, considering the data generated after the emergence of the USA300 clone in the United States during the 2000s, it is crucial to acknowledge the potential rise not only in the severity of infections but also in their overall incidence. This underscores the importance of vigilant surveillance, robust infection control measures, and prompt intervention strategies to mitigate the impact of such scenario (21).

Furthermore, given the potential impact on young, healthy individuals in their productive years, the economic consequences of CA-MRSA infections could be even more substantial. In addition to the direct healthcare costs associated, there may be indirect costs related to lost productivity, absenteeism from work or school, and long-term disability. Therefore, effective prevention and control measures are essential not only for public health but also for maintaining economic stability and productivity in affected communities (22).

From the clinical point of view, physicians must maintain vigilance for these clinical presentations, familiarize themselves with CA-MRSA infections, and be prepared to adjust empirical therapies when necessary. It is noteworthy that while empirical therapy did not initially cover MRSA in the first two cases described in this article, the approach improved in the two subsequent cases, which reflect the adjustment of the treatments in consideration of the previous cases. Importantly, in developing countries, such as Chile, rapid laboratory techniques for early diagnosis of resistance mechanisms are not widely available. Therefore, the selection of empirical therapies continues to rely on physician judgment as long as this situation persists. This underscores the importance of ongoing education and awareness efforts among healthcare professionals to ensure timely recognition and appropriate management of CA-MRSA infections.

In terms of molecular epidemiology, the population structure of *S. aureus* is characterized by high diversity and dynamism, evident in both nosocomial and community-acquired infections. This diversity reflects the complex interplay of diverse factors, including genetic evolution, transmission dynamics, host-pathogen interactions, and environmental influences. Understanding and monitoring this molecular diversity are essential for effective infection control measures, treatment strategies, and public health interventions aimed at mitigating the spread and impact of *S. aureus* infections (23). Considering the above, it has been observed that certain *S. aureus* clones are replace by others, emerging as new dominant lineages in a highly dynamic process. Consequently, this dynamic clonal replacement gives rise to a profoundly heterogeneous global epidemiological landscape. Factors contributing to this complexity include the emergence of novel genetic variants, selective pressures from antimicrobial use, changes in healthcare practices, population movements, and adaptation to different ecological niches. Understanding these dynamics is crucial for effectively managing and controlling *S. aureus* infections on a global scale (3). Accordingly, a notable illustration of this dynamism is the emergence of CA-MRSA infections in the United States during the 1990s. Initially, the USA400 clone was predominant in these infections. However, it was subsequently displaced by one of the most successful clones described to date, namely USA300. This replacement underscores the fluid nature of *S. aureus* epidemiology and highlights the capacity of certain clones to outcompete others and become dominant lineages over time (6). The factors driving this phenomenon are not fully elucidated; however, a range of aspects associated with the microorganism, the environment, and human activity appear to play a relevant role. These factors include the acquisition of a smaller SCC*mec* chromosomal cassette compared to the classic SCC*mec* element, which may enhance bacterial fitness. Additionally, the acquisition of certain virulence factors, such as the arginine catabolic mobile element (ACME) in the USA300 clone, may promote colonization and contribute to the success of specific clones in causing infections. Furthermore, antibiotic resistance genes and heavy metal resistance determinants, such as the copper and mercury resistance (COMER) element found in the Latin American variant of USA300, also contribute to the factors influencing the emergence of specific lineages. These genetic elements confer resistance to antimicrobial agents and environmental stresses, thereby enhancing the adaptability and survival of certain strains in diverse ecological niches (7, 24, 25). Additionally, population migration has been identified as a factor facilitating the introduction of successful clones into new geographic regions. Population movements can lead to the spread of particular strains to previously unaffected areas, thereby contributing to the dissemination and establishment of specific lineages in different populations and geographical locations (26). This phenomenon underscores the interconnectedness of global health and highlights the importance of considering migration patterns in understanding the epidemiology and transmission dynamics of infectious diseases like *S. aureus*.

Furthermore, our analysis revealed that all CA-MRSA strains described in this study belong to the ST8-SCC*mec* IV lineage. This lineage is frequently associated with community-acquired infections globally, with a notable prevalence in the American continent. This observation underscores the widespread distribution and clinical significance of the ST8-SCC*mec* IV lineage in the context of CA-MRSA infections (3, 7, 27). In this context, the primary lineages of community-acquired methicillin-resistant CA-MRSA are the USA300 and its Latin American variant (USA300-LV). These lineages share a common ancestor and have exhibited extensive dissemination. Notably, these clones have demonstrated remarkable success in various geographic regions, including North America and northern South America, specifically Colombia, Venezuela, and Ecuador. This widespread distribution underscores the adaptability and virulence of these lineages across diverse populations and geographical settings (24). One distinguishing feature of USA300-LV is the presence of the genetic element COMER, which confers resistance to copper and mercury that is absent in USA300. Consequently, the presence of the COMER element in USA300-LV may play a significant role in its dissemination and stability, potentially contributing to its success in diverse geographic areas. The ability to withstand environmental stresses, such as heavy metal exposure, could enhance the survival and persistence of USA300-LV in various ecological niches, thereby influencing its spread and establishment in different populations (24).

The migration of populations from regions with a high prevalence of the USA300-LV variant is identified as a potential contributing factor to its spread. This migration pattern may significantly impact the epidemiology of CA-MRSA infections in Chile, particularly due to the substantial migratory movement within the country.^7^ As individuals move between countries, they may carry USA300-LV strains with them, introducing these strains into new regions and potentially leading to localized outbreaks or sustained transmission in the receiving populations. However, further epidemiological studies involving a larger number of strains are necessary to more clearly delineate which clones are predominant in the region and to comprehensively understand the evolutionary dynamics of the process. This holistic approach is essential for informing targeted interventions, enhancing surveillance efforts, and effectively managing MRSA infections in the region.

In terms of molecular epidemiology, all isolates were identified as belonging to the ST8 lineage. However, strains 004UDEC and 008UDEC lack the arginine catabolic mobile element (ACME) and copper and mercury resistance (COMER) elements. These elements are typically harbored by the highly successful USA300 and USA300-LV international clones, respectively. This distinction underscores the genetic diversity within the ST8 lineage and suggests potential differences in virulence and resistance profiles among the strains observed in this study (24). Furthermore, strains 004UDEC and 008UDEC were found to contain the SCC*mec* IVb element, which differs from the SCC*mec* elements normally associated with USA300 and USA300-LV, as well as from other Chilean isolates included in the study. These findings suggest that these isolates are diverging from the predominant clones observed in the region. This divergence highlights the dynamic nature of MRSA, reinforcing the relevance of surveillance and their potential implications for public health.

In the case of the 005UDEC and 014UDEC isolates, they were found to lack the ACME and possess COMER element. This genotype aligns with the characteristic profile of the USA300-LV clone (24), which underscores the relationship of these isolates and the aforesaid clone. However, there are several noteworthy features to highlight: both isolates harbor an SCC*mec* IVb element, which differs from the SCC*mec* IVc element present in the USA300-LV clone. Additionally, the resistome of both isolates is broader than that of the USA300-LV clone (Supplementary Figure S1). Furthermore, the *spa*-type of the 014UDEC isolate could not be assigned by the scheme. All these characteristics suggest that the isolates included in our study might be influenced by evolutionary processes diverging from the well USA300-LV clone. Moreover, all four isolates are not related to the USA300 clone, which differs from the initial description of CA-MRSA in Chile (11).

In terms of the resistome, all isolates were positive for the *blaZ* penicillinase gene, a common genetic determinant widely described in *S. aureus* isolates worldwide (28). Additionally, methicillin resistance in the four isolates was mediated by the presence of the *mecA* gene. Notably, all isolates, including the previously sequenced Chilean strains as well as USA300 and USA300-LV, harbored the *fosB* gene, which confers resistance to fosfomycin (29). In this regard, the *fosB* gene has been identified in numerous MRSA genomes, encompassing both hospital- and community-acquired strains. Its presence contributes to the multidrug-resistant (MDR) phenotype typically exhibited by MRSA isolates (29). Furthermore, all four isolates contained the *tet* (38) gene, which encodes an efflux pump associated with resistance to tetracyclines. However, this gene has also been implicated in virulence, particularly in *S. aureus*’ capacity to internalize and replicate within human epithelial cells (30). Additionally, the 005UDEC isolate harbors two aminoglycoside-modifying enzymes. While aminoglycosides may not currently be a therapeutic option, the presence of these enzymes contributes to the multidrug-resistant (MDR) phenotype exhibited by this isolate.

Regarding virulence genes, all four isolates exhibited a broad virulome, encompassing genes associated with various aspects of pathogenesis. This includes genes involved in adherence, evasion of the immune system, siderophore production (e.g., iron siderophores), enterotoxins, and notably, the production of exotoxins/exoenzymes. Among these latter genes, it is noteworthy to highlight the presence of *geh, hlb, hld, hlgABC, hly/hla, hysA, adsA, aur,* and *pvl* genes in all isolates. These genes contribute significantly to the virulence of the strains, underscoring their potential to cause severe infections and highlighting the importance of understanding their presence in the context of clinical management and public health interventions.

The fours cases described in this case series underscore the importance of maintaining a local and current understanding of the epidemiology of staphylococcal infections in various regions. Such knowledge directly influences clinical decisions, particularly regarding the selection of empirical antibiotic therapy. In regions with low incidence of CA-MRSA, medical teams must remain vigilant and prepared for a potential rise in infections caused by these strains. Therefore, it is imperative to establish comprehensive surveillance programs to gather robust epidemiological data, a practice that is currently lacking in many countries across South America. These programs are essential for early detection, effective management, and prevention strategies against emerging infectious threats.

In summary, since its emergence, the significance of CA-MRSA infections has varied across different regions globally. In some countries, these clones have been established in communities for decades, exerting varying impacts on the populations of these regions and extending into new environments such as hospitals, where they cause nosocomial infections. Conversely, there are countries where the relative importance of CA-MRSA has been lower, such as Chile. However, our work highlights the potential for the emergence of community-acquired infections caused by CA-MRSA lineages in such regions. Therefore, public health systems should be prepared to detect and manage severe and potentially fatal infections caused by these strains effectively.

## Data availability statement

The original contributions presented in the study are publicly available. This data can be found here in the NCBI under BioSample accession numbers: SAMN41135293, SAMN41135294, SAMN41135295, and SAMN41135296.

## Ethics statement

The studies involving humans were approved by Comité de Ética del Servicio de Salud Concepción, Chile. The studies were conducted in accordance with the local legislation and institutional requirements. The participants provided their written informed consent to participate in this study. Written informed consent was obtained from the individual(s) for the publication of any potentially identifiable images or data included in this article.

## Author contributions

AA-R: Conceptualization, Data curation, Formal analysis, Funding acquisition, Investigation, Methodology, Writing – original draft, Writing – review & editing. FM-L: Conceptualization, Data curation, Writing – original draft, Writing – review & editing. MQ-A: Conceptualization, Writing – original draft, Writing – review & editing. MQ: Writing – original draft, Writing – review & editing. SM: Conceptualization, Formal analysis, Writing – original draft, Writing – review & editing. GR: Writing – original draft, Writing – review & editing. NH-C: Investigation, Writing – original draft, Writing – review & editing. YE: Writing – original draft, Writing – review & editing. GG-R: Writing – original draft, Writing – review & editing. AO-C: Conceptualization, Data curation, Methodology, Validation, Visualization, Writing – original draft, Writing – review & editing.

## Footnotes

^1^ https://www.ispch.cl/sites/default/files/BoletinStahylococcusResistente-20062018A%20(1).pdf

^2^ https://www.seqcenter.com/

^3^Available at: https://cge.food.dtu.dk/services/SCCmecFinder/.

^4^Accessible at: https://cge.food.dtu.dk/services/spaTyper/.

^5^ https://usegalaxy.org.au

^6^ https://pathogen.watch/

^7^ https://www.ine.gob.cl/docs/default-source/demografia-y-migracion/publicaciones-y-anuarios/migraci%C3%B3n-internacional/estimaci%C3%B3n-poblaci%C3%B3n-extranjera-en-chile-2018/estimaci%C3%B3n-poblaci%C3%B3n-extranjera-en-chile-2021-resultados.pdf?sfvrsn=d4fd5706_6

## Supplementary material

Supplementary Figure S1 Core genome SNP-base phylogeny of ST8 CA-MRSA isolates. In red are highlighted the four isolates recovered from the clinical cases of this study. Reference genomes used were ST8 USA300 and ST8 USA300-LV (Latin American variant) (accession numbers NKCW01000010.1 and CP007672.1, respectively). 10 Chilean ST8 MRSA isolates previously deposited in the Pathogen Watch database were included. Phylogenetic tree was visualized using the online Interactive Tree of Life (iTOL) (version v6.0) and edited utilizing Inkscape software (version 1.2). COMER, copper, and mercury resistance; ACME, arginine catabolic mobile element; SCC*mec*, Staphylococcal Cassette Chromosome *mec*.

## References

[1] Tong SYC Davis JS Eichenberger E Holland TL Fowler VG. *Staphylococcus aureus* infections: epidemiology, pathophysiology, clinical manifestations, and management. Clin Microbiol Rev. (2015) 28:603–61. doi: 10.1128/CMR.00134-14, PMID: 26016486 PMC4451395

[2] Tacconelli E Carrara E Savoldi A Harbarth S Mendelson M Monnet DL. Discovery, research, and development of new antibiotics: the WHO priority list of antibiotic-resistant bacteria and tuberculosis. Lancet Infect Dis. (2018) 18:318–27. doi: 10.1016/S1473-3099(17)30753-3, PMID: 29276051

[3] Lakhundi S Zhang K. Methicillin-resistant *Staphylococcus aureus*: molecular characterization, evolution, and epidemiology. Clin Microbiol Rev. (2018) 31:20. doi: 10.1128/CMR.00020-18, PMID: 30209034 PMC6148192

[4] Stefani S Chung DR Lindsay JA Friedrich AW Kearns AM Westh H. Meticillin-resistant *Staphylococcus aureus* (MRSA): global epidemiology and harmonisation of typing methods. Int J Antimicrob Agents. (2012) 39:273–82. doi: 10.1016/j.ijantimicag.2011.09.030, PMID: 22230333

[5] Herold BC Immergluck LC Maranan MC Lauderdale DS Gaskin RE Boyle-Vavra S. Community-acquired methicillin-resistant *Staphylococcus aureus* in children with no identified predisposing risk. JAMA. (1998) 279:593–8. doi: 10.1001/jama.279.8.593, PMID: 9486753

[6] Chambers HF DeLeo FR. Waves of resistance: *Staphylococcus aureus* in the antibiotic era. Nat Rev Microbiol. (2009) 7:629–41. doi: 10.1038/NRMICRO2200, PMID: 19680247 PMC2871281

[7] David MZ Daum RS. Community-associated methicillin-resistant *Staphylococcus aureus*: epidemiology and clinical consequences of an emerging epidemic. Clin Microbiol Rev. (2010) 23:616–87. doi: 10.1128/CMR.00081-09, PMID: 20610826 PMC2901661

[8] Ribeiro A Dias C Silva-Carvalho MC Berquó L Ferreira FA Santos RNS. First report of infection with community-acquired methicillin-resistant *Staphylococcus aureus* in South America. J Clin Microbiol. (2005) 43:1985–8. doi: 10.1128/JCM.43.4.1985-1988.2005, PMID: 15815039 PMC1081335

[9] Arias CA Reyes J Carvajal LP Rincon S Diaz L Panesso D. A prospective cohort multicenter study of molecular epidemiology and phylogenomics of *Staphylococcus aureus* bacteremia in nine Latin American countries. Antimicrob Agents Chemother. (2017) 61:816. doi: 10.1128/AAC.00816-17, PMID: 28760895 PMC5610503

[10] Corso A Sanches IS De Sousa MA Rossi A De Lencastre H. Spread of a methicillin-resistant and multiresistant epidemic clone of *Staphylococcus aureus* in Argentina. Microb Drug Resist. (1998) 4:277–88. doi: 10.1089/MDR.1998.4.277, PMID: 9988046

[11] Noriega LM González P Hormazábal JC Pinto C Canals M Munita JM. *Staphylococcus aureus* comunitario resistente a cloxacilina: Comunicación de los primeros cinco casos descritos en Chile community acquired infections with methicillin resistant strains of *Staphylococcus aureus*. Report of five cases. Rev Med Chile. (2008) 136:885–91. doi: 10.4067/S0034-98872008000700010 18949165

[12] Page AJ Taylor B Delaney AJ Soares J Seemann T Keane JA. *SNP*-sites: Rapid efficient extraction of SNPs from multi-FASTA alignments. Microb Genom. (2016) 2:e000056. doi: 10.1099/mgen.0.000056 28348851 PMC5320690

[13] Letunic I Bork P. Interactive tree of life (iTOL) v5: an online tool for phylogenetic tree display and annotation. Nucleic Acids Res. (2021) 49:W293–6. doi: 10.1093/nar/gkab301, PMID: 33885785 PMC8265157

[14] Mohseni M Rafiei F Allah Ghaemi E. (2018) High frequency of exfoliative toxin genes among *Staphylococcus aureus* isolated from clinical specimens in the north of Iran: Alarm for the health of individuals under risk. Available at: http://ijm.tums.ac.ir (Accessed March 21, 2024). PMC6087700 30112153

[15] Medina G Egea AL Otth C Otth L Fernández H Bocco JL. Molecular epidemiology of hospital-onset methicillin-resistant *Staphylococcus aureus* infections in southern Chile. Eur J Clin Microbiol Infect Dis. (2013) 32:1533–40. doi: 10.1007/s10096-013-1907-8, PMID: 23765159

[16] Malcolm B. The rise of methicillin-resistant *Staphylococcus aureus* in U.S. correctional populations. J Correct Health Care. (2011) 17:254–65. doi: 10.1177/1078345811401363, PMID: 21571749 PMC3116074

[17] Hunt C Dionne M Delorme M Murdock D Erdhich A Wolsey D. Four pediatric deaths from community-acquired methicillin-resistant *Staphylococcus aureus*—Minnesota and North Dakota, 1997-1999. JAMA. (1999) 282:1123–5. doi: 10.1001/JAMA.282.12.1123-JWR0922-2-1, PMID: 10501104

[18] Löffler B Niemann S Ehrhardt C Horn D Lanckohr C Lina G. Pathogenesis of *Staphylococcus aureus* necrotizing pneumonia: the role of PVL and an influenza coinfection. Expert Rev Anti-Infect Ther. (2013) 11:1041–51. doi: 10.1586/14787210.2013.827891, PMID: 24073746

[19] Niemann S Ehrhardt C Medina E Warnking K Tuchscherr L Heitmann V. Combined action of influenza virus and *Staphylococcus aureus* Panton–valentine Leukocidin provokes severe lung epithelium damage. J Infect Dis. (2012) 206:1138–48. doi: 10.1093/INFDIS/JIS468, PMID: 22837490 PMC3433859

[20] Voyich JM Otto M Mathema B Braughton KR Whitney AR Welty D. Is Panton-valentine Leukocidin the major virulence determinant in community-associated methicillin-resistant *Staphylococcus aureus* disease? J Infect Dis. (2006) 194:1761–70. doi: 10.1086/509506, PMID: 17109350

[21] Carrel M Perencevich EN David MZ. USA300 methicillin-resistant *Staphylococcus aureus*, United States, 2000–2013. Emerg Infect Dis. (2015) 21:1973–80. doi: 10.3201/EID2111.150452, PMID: 26484389 PMC4622244

[22] Lee BY Singh A David MZ Bartsch SM Slayton RB Huang SS. The economic burden of community-associated methicillin-resistant *Staphylococcus aureus* (CA-MRSA). Clin Microbiol Infect. (2013) 19:528–36. doi: 10.1111/j.1469-0691.2012.03914.x, PMID: 22712729 PMC3463640

[23] Bal AM Coombs GW Holden MTG Lindsay JA Nimmo GR Tattevin P. Genomic insights into the emergence and spread of international clones of healthcare-, community-and livestock-associated meticillin-resistant *Staphylococcus aureus*: blurring of the traditional definitions. J Glob Antimicrob Resist. (2016) 6:95–101. doi: 10.1016/j.jgar.2016.04.004, PMID: 27530849

[24] Planet PJ Diaz L Kolokotronis S-O Narechania A Reyes J Xing G. Parallel epidemics of community-associated methicillin-resistant *Staphylococcus aureus* USA300 infection in north and South America. J Infect Dis. (2015) 212:1874–82. doi: 10.1093/infdis/jiv320, PMID: 26048971 PMC4655856

[25] Otto M. Community-associated MRSA: what makes them special? Int J Med Microbiol. (2013) 303:324–30. doi: 10.1016/J.IJMM.2013.02.007, PMID: 23517691 PMC3729626

[26] Stegger M Wirth T Andersen PS Skov RL De Grassi A Simões PM. Origin and evolution of European community-acquired methicillin- resistant *Staphylococcus aureus*. MBio. (2014) 5:1044–58. doi: 10.1128/MBIO.01044-14 PMC4173770 25161186

[27] Arias CA Rincon S Chowdhury S Martínez E Coronell W Reyes J. MRSA USA300 clone and VREF—A U.S.–Colombian connection? N Engl J Med. (2008) 359:2177–9. doi: 10.1056/NEJMC0804021, PMID: 19005205 PMC2762734

[28] Aguayo-Reyes A Quezada-Aguiluz M Mella S Riedel G Opazo-Capurro A Bello-Toledo H. Bases moleculares de la resistencia a meticilina en *Staphylococcus aureus*. Rev Chilena Infectol. (2018) 35:7–14. doi: 10.4067/s0716-10182018000100007, PMID: 29652966

[29] Monte DFM de Oliveira CJB. Global trends in the increasing prevalence of the fosfomycin resistance gene in *Staphylococcus aureus*. Lancet Microbe. (2024) 5:e104. doi: 10.1016/S2666-5247(23)00339-7, PMID: 37956689

[30] Truong-Bolduc QC Bolduc GR Medeiros H Vyas JM Wang Y Hooper DC. Role of the Tet 38 efflux pump in *Staphylococcus aureus* internalization and survival in epithelial cells. Infect Immun. (2015) 83:4362–72. doi: 10.1128/IAI.00723-15, PMID: 26324534 PMC4598397

