# Supplementary figures and images for: Community-acquired methicillin-resistant Staphylococcus aureus invasive infections: a case series from Central-South Chile
Source: Front Med (Lausanne). 2024 May 15;11:1365756. doi: 10.3389/fmed.2024.1365756 (PMC11133615; doi:10.3389/fmed.2024.1365756)

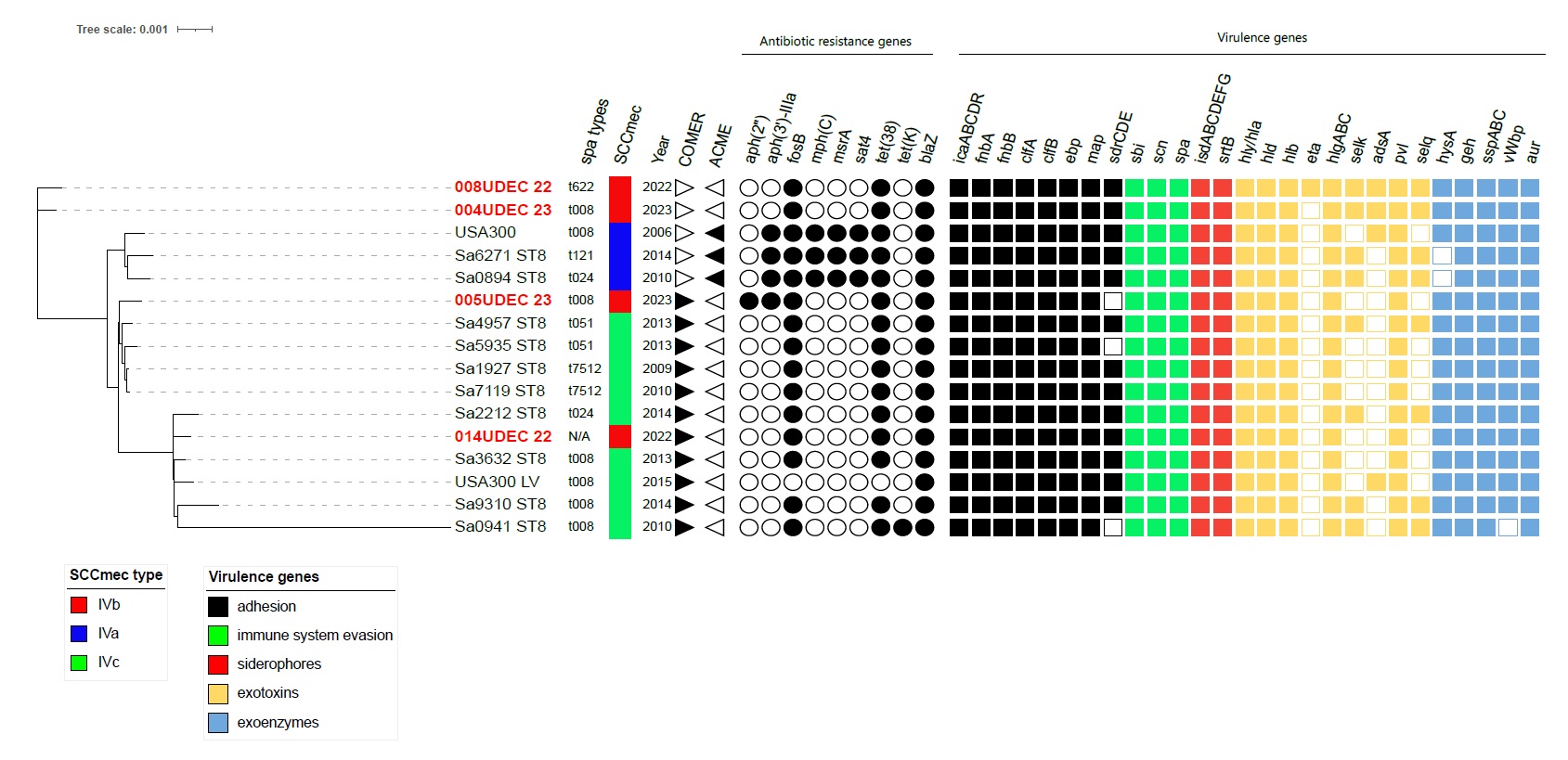

Supplement: Supplementary Figure S1 — Core genome SNP-base phylogeny of ST8 CA-MRSA isolates. In red are highlighted the four isolates recovered from the clinical cases of this study. Reference genomes used were ST8 USA300 and ST8 USA300-LV (Latin American variant) (accession numbers NKCW01000010.1 and CP007672.1, respectively). 10 Chilean ST8 MRSA isolates previously deposited in the Pathogen Watch database were included. Phylogenetic tree was visualized using the online Interactive Tree of Life (iTOL) (version v6.0) and edited utilizing Inkscape software (version 1.2). COMER, copper, and mercury resistance; ACME, arginine catabolic mobile element; SCCmec, Staphylococcal Cassette Chromosome mec. [file Image_1.JPEG]
